# 

**Published:** 1890-06

**Authors:** 


### INDEX TO VOL. V.—JULY, 1889, TO JUNE, 1890.

A Breach of Ethics .... 32
A Remarkable Case of Elephantiasis .... 52
A Tarantula Bite .... 53
A Portable Gynecological Table .... 138
A Case of Triplets .... 330
Acute Gastritis in Children .... 222
A Second Sarah .... 259
A Week of Practice in Mexico .... 332
Address of Retiring President, West Tex. Med. Soc .... 226
Artificial Respiration; an Improved Method .... 59
Book Notices .... 23, 197, 275, 357, 393, 477
  American Armanentarium Chirurgicum .... 153
  Bright's Disease, Lectures on .... 197
  Favorite Prescriptions of Distinguished Practitioners .... 153
  Keating's Cyclopedia of Diseases of Children, Volumes I and II .... 72, 152
  Sweedish Movement and Massage .... 197
  Text Book, Medical Jurisprudence .... 154
  Transactions, Texas State Medical Association .... 150, 198
Berrey, Dr., Letter From .... 36
Blood Letting in Tarantula Bite .... 90
Brown-Sequard's Discovery: The Scientific Aspect of .... 99
Bushwacking .... 63
Biography Contemporary Physicians of Texas .... 144, 236
Cullings from Contemporaries .... 96, 20
Cherokee County Medical Society .... 21
Charter of the Texas State Medical Association .... 13
Carbolic Acid in Mammary Abscess .... 7
Circumcision enforced by Law .... 26
Can Extra Uterine Pregnacy Terminate in Natural Delivery? .... 234
Compound Presentation .... 163
Closure of Both External Auditory Canals .... 432
Digestive Ferments .... 26
Diphtheria; Report of a case .... 81, 103

Diagnosis in Disease....................................329
Death of Dr. J. T. Webb.................................... 93
Dr. Paine's Discoveries in Electro-therapeutics.............145
Dr. Merryman Drops In.................................191
Dr. McLaughlin's New Hemostatic Forceps...............268
Dr. Lowry's Letter: His Case of Extra-Uterine Pregnancy...303
Dr. H. K. Leake, Letter from..............................434
Extra-Uterine Pregnancy...................................258
Extra-Uterine Pregnancy, Was it...........................223
Extra-Uterine Pregnancy, Dr. Lowry's Case...............303
Forceps, Dr. McLaughlin's New Hemostatic................268
Further Success with Nitrate Potassa in Chill and Fever.336,463
Fiat Justitia.................................................474
Gastritis in Children.......................................255
Gunshot Wound of the Bladder,.............................449
Hastening Slowly...........................................442
Infantile Convulsions........................................ 11
Ice; Natural or Artificial...................................... 75
Important to County Societies............................101
Infantile Convulsions........................................108
Is the Influence of the Medical Profession on the Wane?.....263
Important Announcement.................................269
Inhalation in Whooping Cough............................ 87
Life; an Essay....................................280, 321
Ligation of Subclavian, and Carotid Artery.................. 50
Laparatory for Gunshot Wound of the Bladder, ..........449
Medical Legislation, Two Committees on..................444
Malpractice.................................................208
Maternal Impressions on the Fetus in Utero..............201
Microscope, The..........................................206
Milk Inspection in Cities.................................232
Multiple Colloid Cystoma of Left Ovary...................139
Mineral Waters of Texas, ................................453
Minor Surgery............................................. 57
Medical Department, University of Texas..................189
Medical News and Miscellany, 70, 109, 154, 156, 194, 237, 270, 315, 395, 399, 352, 354, 444, 474........................
Mississippi Valley Medical Association..................... 19

Medical Education .................................... 23
Newsy Letter from San Antonio .......................... 16
New and Valuable Instrument of Texas Invention ......... 107
Notes of the Convention ................................ 443
Nitrate of Potassa in Chill ...................... 300, 463
Nitrate of Potassa in Chill, Further Success with .. 336, 463
Official Preparations of Uncertain Strength ............ 311
Portable Gynecological Table, A ........................ 138
Purperal Eclampsia, Report of a Case ................... 166
Puerperal Convulsions .................................. 365
Placenta Prævia ........................................ 171
Preventive Treatment of Urinary Calculi ................ 177
Perityphlitis, with Abscess of Appendix ................ 367
Potassi Nitras in Chill and Fever ...................... 300
Paretic Dementia ....................................... 287
Pharmacy in Mexico ..................................... 96
Perry, Dr. J.; Letter from ............................. 306
Physicians' Mutual Benefit Association ..................
Puerperal Eclampsia .................................... 1
Pin Extracted from a Woman's Throat .................... 14
Quackery Rampant ....................................... 334
Reasons Why Physicians Should Patronize a Southern Poly-clinic ... 392
Raising the Standard of Medical Education .............. 349
Rise and Fall of the P. M. B. A., The .................. 68
Rosser, Dr. C. M., Letter from ......................... 305
Recent Meeting Texas State Medical Association ......... 439
Successful Case of Laparotomy .......................... 383
Some Amusing Theories of By-gone Days .................. 381
Summer Diarrhœa in Children ............................ 83
Some Observations on the Possibility of Preventive Surgery .. 119
Surgical Clinic in Memphis; Hospital Notes ............. 244
Sub-Peritoneal Hematocele .............................. 252
Screw Worms in the Mouth ............................... 384
Similia Similibus; Is it? .............................. 267
SOCIETY NOTES—
Austin District Medical Society ........... 144, 259, 319, 385
Address of President West Texas Medical Society ........ 226

American Medical Association........................346
Arrangement for meeting T. S. M. A...................388
Bosque County Medical Society....................... 95
Central Texas Medical Society.....................60, 262
Cherokee County Medical Society.................342, 467
Death of Dr. Webb, Terrell County Med. Soc........... 93
Johnson County Medical Society......................338
New Tri-State Medical Society....................... 96
Revision U. S. Pharmacopœia.........................387
Southern Surgical and Gynecological Association.....95, 144
Travis County Medical Sosiety....................23, 309
Texas State Medical Association.................308, 345
Tri-State Sanitary Association......................310
Tenth International Medical Congress................340
Terrell County Medical Society..................344, 464
Texas Sanitary Association..........................348
West Texas Medical Association..........92, 94, 185, 262
Taylor County Medical Society.......................437
The Banquet Question..............................89, 91
Trachoma............................................184
The Latest Elixir Vitæ.............................. 65
Texas State Medical Association, New Members........431
" " " 22nd Annual Meeting....308
" " " Proceedings............407
Texas as a Health Resort............................147
Tape Worm...........................................251
Two Committees on Medical Legislation...............444
Two Deaths from Partial Chloroform Anæsthesia........248
The 22nd Annual Meeting Texas State Medical Association..389
The Journals Aim and Mission........................ 29
The Youngest Mother on Record....................... 19
The End of Volume Five..............................472
Unpleasant, but a Duty, Nevertheless................469
Vis Medicatrix Naturæ............................... 43
Williams, Dr. R.G., Letter from.....................308
Where's the Malancholy?.............................149
W. P. Burts, M. D., President Texas State Medical Association.....471
Young Parents....................................... 59

